# FMRP Levels in Human Peripheral Blood Leukocytes Correlates with Intellectual Disability

**DOI:** 10.3390/diagnostics11101780

**Published:** 2021-09-28

**Authors:** Mark Roth, Lucienne Ronco, Diego Cadavid, Blythe Durbin-Johnson, Randi J. Hagerman, Flora Tassone

**Affiliations:** 1 Fulcrum Therapeutics, Cambridge, MA 02139, USA; lucienne.ronco@gmail.com (L.R.); cadavid.diego@gmail.com (D.C.); 2 Division of Biostatistics, University of California Davis, School of Medicine, Davis, CA 95616, USA; bpdurbin@ucdavis.edu; 3 Department of Pediatrics, University of California Davis, School of Medicine, Sacramento, CA 95616, USA; rjhagerman@ucdavis.edu; 4 MIND Institute, University of California Davis Medical Center, Sacramento, CA 95817, USA; 5 Department of Biochemistry and Molecular Medicine, University of California Davis, School of Medicine, Sacramento, CA 95817, USA

**Keywords:** Fragile X syndrome, *FMR1*, FMRP, qRT-PCR, PrimeFlow^TM^, MSD, PBMCs, IQ

## Abstract

Fragile X syndrome (FXS) is the most common form of inherited intellectual disability. FXS is an X-linked, neurodevelopmental disorder caused by a CGG trinucleotide repeat expansion in the 5′ untranslated region (UTR) of the Fragile X Mental Retardation gene, *FMR1*. Greater than 200 CGG repeats results in epigenetic silencing of the gene leading to the deficiency or absence of Fragile X mental retardation protein (FMRP). The loss of FMRP is considered the root cause of FXS. The relationship between neurological function and FMRP expression in peripheral blood mononuclear cells (PBMCs) has not been well established. Assays to detect and measure *FMR1* and FMRP have been described; however, none are sufficiently sensitive, precise, or quantitative to properly characterize the relationships between cognitive ability and CGG repeat number, *FMR1* mRNA expression, or FMRP expression measured in PBMCs. To address these limitations, two novel immunoassays were developed and optimized, an electro-chemiluminescence immunoassay and a multiparameter flow cytometry assay. Both assays were performed on PMBCs isolated from 27 study participants with *FMR1* CGG repeats ranging from normal to full mutation. After correcting for methylation, a significant positive correlation between CGG repeat number and *FMR1* mRNA expression levels and a significant negative correlation between FMRP levels and CGG repeat expansion was observed. Importantly, a high positive correlation was observed between intellectual quotient (IQ) and FMRP expression measured in PBMCs.

## 1. Introduction

Fragile X syndrome (FXS) is one of the *FMR1*-associated conditions characterized by a broad spectrum of intellectual and cognitive deficits, including a large constellation of behavioral and physical features [1,2,3]. These deficits are primarily attributed to the loss of FMRP encoded by the *FMR1* gene. The underlying causative mutation, in almost all FXS patients, is the expansion of greater than 200 CGG trinucleotide repeats, located in the 5′ UTR of the *FMR1* gene. The CGG expansion leads to hypermethylation, transcriptional silencing, and consequent absence of FMRP, a key modulator of neuronal synaptic plasticity and dendritic morphology [4]. Cellular dysregulation related to the loss of FMRP involves multiple pathways important for neurological function. A recent report demonstrated that an overall expression of greater than 20% normal is sufficient to normalize neuronal hyperactivity in FXS iPSC-derived neurons [5].

FMRP is widely expressed, with particularly high expression in neurons and testis [6,7]. FMRP affects numerous signaling pathways through its interaction with RNAs and proteins. The affected pathways include DNA and/or RNA regulation; DNA damage repair, mRNA splicing, editing, and trafficking; and channel binding and protein synthesis suppression [8]. 

*FMR1* CGG repeat expansion sizes fall into three broad categories: normal (<55 CGG repeats), premutation (55 to 200 CGG repeats), and full mutation (>200 CGG repeats). The estimated prevalence of the premutation ranges from 1 in 110 to 1 in 209 for females and from 1 in 430 to 1 in 813 for males [9]. While FXS is caused by a full mutation of the *FMR1* gene, individuals carrying premutations are at risk for developing *FMR1* associated disorders such as Fragile X-associated tremor/ataxia syndrome [10] (FXTAS), which leads to neurological impairment in elderly adults; Fragile X-associated primary ovarian insufficiency [11,12] (FXPOI), which can lead to infertility and/or early menopause in approximately 16% of female premutation carriers; and Fragile X-associated neuropsychiatric disorders [13] (FXAND), which includes anxiety, depression, ADHD, social deficits, and autism spectrum disorders (ASD). In addition, individuals with the premutation can experience a wide variety of other clinical problems, including sleep apnea, hypertension, immune-mediated conditions, seizure, neuropathy, fibromyalgia, migraine, psychiatric conditions, and chronic fatigue [14,15].

In contrast to FXS, where the full mutation results in lack of FMRP, premutation-associated disorders are believed to be caused by RNA toxicity, as there is a CGG dependent, 2- to 10-fold increase in *FMR1* levels, with normal or moderate reduction of FMRP expression [16,17,18]. Individuals that carry either the full mutation or the premutation can be mosaic for allele size or methylation [19,20,21], meaning they can carry alleles of different CGG repeat size and/or amount of allelic methylation. Specifically, in these individuals FMRP expression is reduced because some alleles are methylated and therefore transcriptionally silent, while others can be unmethylated but not efficiently translated due to the larger CGG repeat length.

A few studies have investigated and reported on the relationship between FMRP levels and intellectual quotient (IQ) [22,23] mainly in peripheral tissues or cultured cell lines using various methodologies. The different methodologies described to measure FMRP levels in cell or tissue types such as peripheral blood mononuclear cells (PBMCs), platelets, cell lines, fibroblasts, and brain over the past ten to fifteen years include immunohistochemistry, Western blot, ELISA, and fluorescence resonance energy transfer (FRET) assays. While these techniques have moved the field forward, they have important limitations. These assays tend to be semiquantitative, labor intensive, and/or work poorly when using PBMCs. Thus, there remains a high unmet need for robust methodologies to accurately quantify FMRP expression in human cells, specifically in PBMCs.

Accurate measurement of FMRP expression is important to study the correlations between the FXS clinical phenotypes and the expression levels of FMRP in cells or tissues accessible for repeated testing such as PBMCs. Repeated, accurate measurements of FMRP from accessible tissue could prove valuable in proof-of-concept studies of novel therapies to reactivate FMRP expression. In addition, FMRP levels themselves may be considered for use as an outcome measure in clinical trials. In this study, two novel assays to measure FMRP and/or *FMR1* mRNA directly in PBMCs were developed. The first assay is a quantitative, high throughput, electrochemiluminescence assay [24,25] with a low false detection rate, a 0.07 fmol lower limit of detection (LLOD), and a 0.65 fmol lower limit of quantification (LLOQ). The second assay, based on PrimeFlow^TM^ by ThermoFisher Scientific (Waltham, MA, USA) [26,27], is a flow cytometric assay which simultaneously immunophenotypes PMBCs and measures the relative amounts of *FMR1* and FMRP. 

Both assays were applied to PMBCs isolated from male participants with a broad spectrum of *FMR1* mutations, covering a wide range of CGG repeat and methylation status. The levels of *FMR1* mRNA and FMRP expression were correlated with IQ. The goals of this study were to (1) determine the relationship between CGG repeat number and *FMR1* mRNA and FMRP expression in PBMCs after correcting for methylation status, (2) determine the change in *FMR1* mRNA and FMRP expression as the number of CGG repeats increased from normal to full mutation, and (3) determine if a relationship exists between FMRP expression in PBMCs and IQ. Our findings show that FMRP levels in PBMCs were inversely correlated with CGG repeat length, and most importantly, PBMC FMRP levels were highly correlated to IQ. 

## 2. Materials and Methods

### 2.1. Study Participants

In total, 27 male participants, 23 with a broad spectrum of *FMR1* mutations covering the premutation and full mutation range, and 4 controls carrying an allele in the normal range were included in this study (Table 1). Ages ranged from 3 to 74 years for the cases and from 52 to 72 years for the controls, respectively. The study was carried out in accordance with the Institutional Review Board (IRB: 1094641-2) at the University of California (Project identification code: 1094641-2), Davis with written informed consent obtained from all participants in accordance with the Declaration of Helsinki.

### 2.2. CGG Repeat Sizing and Methylation Status

Genomic DNA (gDNA) was isolated from 5 mL of peripheral blood leukocytes using the Gentra Puregene Blood Kit (Qiagen, Valencia, CA, USA) and used for PCR and Southern Blot analysis as previously reported [28,29]. Methylation status was assessed by Southern Blot analysis as described in Tassone et al., 1999 [22] (Table 1).

### 2.3. FMR1 mRNA Expression Levels

Total RNA was isolated from 2.5 mL of peripheral blood collected in PAXgene Blood RNA tubes using the PAXgene Blood RNA Kit (Qiagen, Valencia, CA, USA). Total RNA concentration was measured using the Agilent 2100 Bioanalyzer system. cDNA synthesis and real-time PCRs (qRT-PCR), performed using Assays-On-Demand from Applied Biosystems (Applied Biosystems, Foster City, CA, USA) were as previously reported [16] and measurements were carried out in three concentrations in duplicate for each sample [16] (Table 1).

### 2.4. Frozen PBMC Preparation for Electrochemiluminescence and PrimeFlow^TM^ Assays

Peripheral blood was collected in Cell Preparation Tube (CPT) vacutainers with sodium citrate (Becton Dickinson) and centrifuged according to the manufacturer’s recommendations for separating mononuclear cells from whole blood. PBMCs were washed with Dulbecco’s phosphate buffered saline (PBS) and frozen in RPMI 1640 media with 10% fetal bovine serum and 10% dimethyl sulfoxide. Frozen, isolated PBMCs were quickly thawed in a 37 °C water bath. The thawed PBMCs were diluted into 10 mL of flow buffer (PBS, 5% FBS, 2 mM EDTA, 0.09% sodium azide). The suspension was passed through a 70 μM filter (Miltenyi Biotech, Auburn, CA, United States) into a fresh tube. The cellularity of the filtered suspension was counted on a ThermoFisher Countess (ThermoFisher Scientific, Waltham, MA, USA). Cells were pelleted (800× *g* for 7 min) and the supernatant aspirated. The cells were resuspended in a flow buffer at 1 × 10^7^ cells/mL. 3 × 10^6^ cells in a total of 0.3 mL were set aside for PrimeFlow^TM^ processing. The remaining cells were washed two times with PBS then lysed in 50 μL RIPA buffer with cOmplete and PhosSTOP added (Millipore-Sigma, St. Louis, MO, USA). Lysates were clarified by centrifugation at 12,000× *g* for 12 min at 4 °C, then the clarified lysates were stored at −80 °C.

### 2.5. PrimeFlow^TM^ Flow Cytometric Assay

PrimeFlow^TM^ was carried out per manufacturer instructions with slight modifications including the addition of surface and intracellular protein staining. 1 × 10^6^ cells of the reserved cell suspension were added to each of 3 Eppendorf tubes supplied with the PrimeFlow^TM^ Kit (ThermoFisher Scientific, Waltham, MA, USA). Cells were incubated 10 min at ambient temperature with Fc Block (Miltenyi Biotech, Auburn, CA, USA). A pre-mixture of CD8a-eFluor 450, CD19-PE/Cyanine 5.5, CD14-PE/Cyanine 7, CD3-APC/eFluor 780, CD4-PE/eFluor 610, and Fixable Viability Dye eFluor 506 was added to each tube (ThermoFisher Scientific, Waltham, MA, USA) and incubated for 30 min at 4 °C. Cells were washed twice with flow buffer. Samples were fixed for 30 min at 4 °C with PrimeFlow^TM^ buffer and washed twice with 1X PrimeFlow^TM^ RNA Permeabilization Buffer containing RNase Inhibitors. 

Anti-FMRP antibody 5C2-Alexa Fluor 488 (Biolegend, San Diego, CA, USA) was added to 2 of the 3 aliquots for each sample. The remaining aliquot was stained with mouse IgG1 isotype control-Alexa Fluor 488 (ThermoFisher Scientific, Waltham, MA, USA). Samples were incubated for 30 min at 4 °C and washed three times with 1X PrimeFlow^TM^ RNA Permeabilization Buffer with RNase Inhibitors. Samples were fixed for 60 min in the dark at ambient temperature using 10X PrimeFlow^TM^ RNA Fixation Buffer 2 and then washed twice with1X PrimeFlow^TM^ RNA Wash Buffer. For each set of 3 Eppendorf tubes, 2 aliquots of *FMR1* mRNA probe (ThermoFisher Scientific, Waltham, MA, USA) and 1 aliquot of dapB mRNA probe (ThermoFisher Scientific, Waltham, MA, USA) were prepared in PrimeFlow^TM^ RNA Target Probe Diluent. One of the *FMR1* mRNA aliquots was added to an FMRP tube and the other *FMR1* mRNA aliquot was added to the isotype control tube. The dapB mRNA aliquot was added to the remaining FMRP tube. All tubes were incubated for 2 h at 40 °C with several inversions after the 1st hour. Samples were washed once with PrimeFlow^TM^ RNA Wash Buffer. Samples were next washed with PrimeFlow^TM^ RNA Wash Buffer + RNase Inhibitors. The samples were resuspended in the residual 100 μL and stored overnight at 4 °C.

The next day PrimeFlow^TM^ RNA PreAMP Mix was added to each tube and incubated 1.5 h at 40 °C. Samples were washed three times in PrimeFlow^TM^ RNA Wash Buffer. PrimeFlow^TM^ RNA AMP Mix was added to each tube and incubated 1.5 h at 40 °C. Samples were washed twice in PrimeFlow^TM^ RNA Wash Buffer. PrimeFlow^TM^ RNA Labeled Probes were diluted 1:100 in PrimeFlow^TM^ RNA Label Probe Diluent and diluted label probes were added to each tube. Samples were incubated 1 h at 40 °C then washed twice with PrimeFlow^TM^ RNA Wash Buffer and once with eBioscience Flow Cytometry Staining Buffer (ThermoFisher Scientific, Waltham, MA, USA). Data was acquired on an ATTUNE NXT cytometer (ThermoFisher Scientific, Waltham, MA, USA). Data was analyzed using FlowJo software (Becton Dickinson, San Jose, CA, USA (Table 1)).

### 2.6. Absolute Quantification of FMRP by Electrochemiluminescence ELISA (MSD)

All antibody and lysate dilutions were done in MSD Diluent 100 (Meso Scale Discovery, Rockville, MD, USA). A PBMC lysate, 150 µg/mL FMRP positive control lysate, 150 µg/mL FMRP negative control lysate, or a standard curve of recombinant FMRP (Origene, Rockville, MD, USA) was mixed 1:1:1 with custom biotinylated rabbit, polyclonal anti-FMRP ab17722, final concentration 1 µg/mL (Abcam, Cambridge, MA, USA) and mouse monoclonal 6B8/FMRP, final concentration 0.5 µg/mL (Biolegend, San Diego, CA, USA) in a V-bottom, polypropylene, 96-well plate. The plate was sealed and placed on a shaker at 4 °C overnight. Overall, 5 μL of the mixture was added to each well of a 384-well avidin coated MSD plate (Meso Scale Discovery, Rockville, MD, USA) in quadruplicate. Mixtures were incubated at ambient temperature for 1 h on a 750 RPM shaker. The plate was washed three times using a 50 μL MSD wash buffer per well. The plate was blocked for 1 h on a 750 RPM shaker at ambient temperature in 3% MSD Blocker A in MSD wash buffer, 40 μL/well. The plate was washed three times using MSD wash buffer. Then, 5 μL sulfo-tagged anti-mouse diluted 1:500 was added to each well of the plate. This was incubated at ambient temperature for 1 h while shaking at 750 RPM. The plate was washed three times using MSD wash buffer. 4X MSD read buffer T (Meso Scale Discovery, Rockville, MD, USA) was diluted in deionized water to give a 2X MSD read buffer T. 40 μL 2X MSD read buffer T was added to each well. The voltage was optimized by the instrument manufacturer. The peak voltage applied during excitation was approximate 5 V. The plate was immediately acquired using the MESO SECTOR S 600 reader. Data was analyzed using MSD software. A standard curve in fmol was created from the recombinant FMRP. The fmol of FMRP for each lysate was calculated from the standard curve. Supplementary Figure S1 illustrates a standard curve and locations of a full mutation, premutation, and full mutation participants with equivalent total protein lysates. Data were reported as fmol FMRP per μg total protein (Table 1).

### 2.7. Total Protein Concentration

PBMC lysate concentrations were below the detection limit of a BCA reaction. Therefore, the highly sensitive ProteinSimple Total Protein Detection Module (ProteinSimple, San Jose, CA, USA) was modified to determine lysate concentrations. A PBMC lysate standard was created from a large batch of isolated PBMCs. The protein concentration of the PBMC standard was determined using the BCA assay (Millipore-Sigma, St. Louis, MO, USA). A 4-point standard lysate curve and lysates from the test PBMCs were prepared following the protocol provided with the Total Protein Detection module (ProteinSimple, San Jose, CA, USA). The prepared lysates were run in the 12–230 kDa separation module (ProteinSimple, San Jose, CA, USA) on the JESS (ProteinSimple, San Jose, CA, USA). Data analysis was performed using Compass software (ProteinSimple, San Jose, CA, USA). For each point on the standard cure, the area under the curve (AUC) for peaks at 48, 75, and 190 kDa was calculated. The AUCs for the same peaks in the sample lysates were determined. The values from the standard curve were used to determine the concentrations of the sample lysates.

### 2.8. IQ Measurements

Cognitive testing was carried out at the time of the visit and of blood sample collection, with standardized IQ measures depending on participants’ age. These included Mullen Scales of Early Learning (MSEL) [30], Weschler abbreviated Scale of Intelligence (WASI) [31], Wechsler Intelligence Scale for Children (WISC-III) [32], Stanford–Binet Intelligence Scales (Stanford Binet V) [33] and The Leiter International Performance Scales–Revised [34] as shown in (Table 1).

### 2.9. Statistical Methods

*FMR1* and FMRP expression were modelled by the number of CGG repeats using multiple linear regression methods. To address methylation mosaicism, percent methylation was adjusted for by including it in the model as a covariate. FMRP was modelled by *FMR1* using a multiple linear regression model with linear and quadratic terms for *FMR1*, which allows FMRP to increase with increasing *FMR1* up to a point (approximately 150 CGG repeats) then decrease. The correlations between FMRP as measured by different methods and *FMR1* mRNA as estimated by different methods were estimated using Pearson. IQ was modelled by FMRP expression using linear regression. Analyses were conducted using R, version 3.6.3 (R Core Team, 2020).

## 3. Results

The laboratory workflow for the execution of the assays carried out in this study using PMBCs is outlined in Supplementary Figure S2. The operator ran and analyzed the MSD and PrimeFlow^TM^ assays blinded to the qRT-PCR *FMR1* expression levels, CGG repeat number data, and participant IQ. Duplicate samples from eight individuals were assayed to test reproducibility of the PrimeFlow^TM^ assay. In all two variable plots, data were graphed only if the values for both variables were determined; therefore, not all plots consist of 27 points.

### 3.1. FMR1 mRNA and FMRP Expression Levels Correlate with CGG Repeat Number

**Figure 1:**
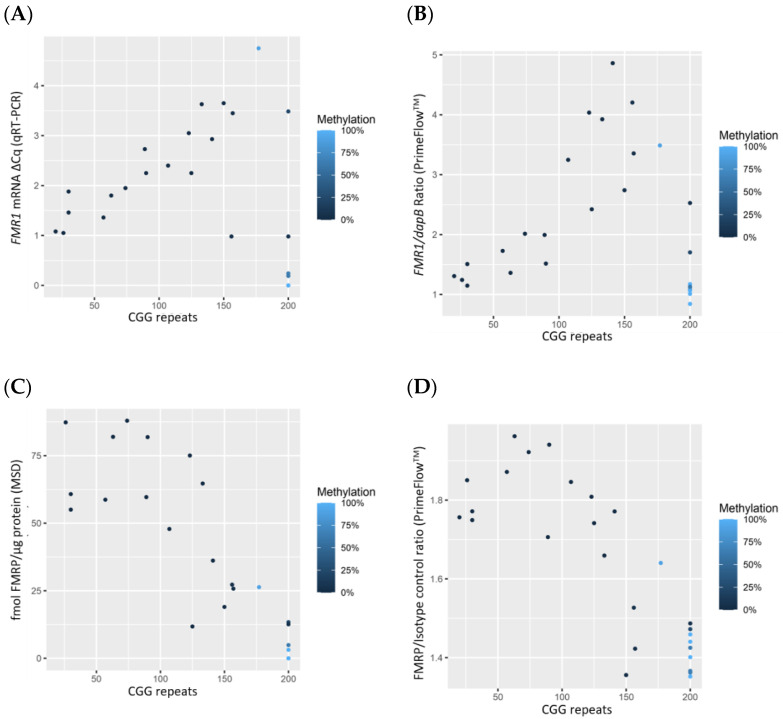
Scatterplots showing a significant positive correlation between CGG repeat length and *FMR1* expression levels measured using qRT-PCR (*n* = 25) (**A**) and PrimeFlow^TM^ (*n* = 27) (**B**). Scatterplot of CGG repeat length versus FMRP expression measured by MSD (*n* = 24) (**C**) and by PrimeFlow^TM^ (*n* = 27) (**D**). Points are colored by % methylation, with lighter blue corresponding to more methylation and darker blue to less methylation (see Table 1). A small amount of random jitter is applied to the x-axis in order to better display overlapping points.

As expected for both mRNA measurements, the qRT-PCR and PrimeFlow^TM^ assays, a positive correlation was observed between *FMR1* mRNA expression levels and CGG repeat number in participants with fewer than 200 CGG repeats after correcting for percentage of methylation (qRT-PCR *p* = 0.001, covariate adjusted correlation = 0.63, and the PrimeFlow^TM^ assays *p* < 0.001, covariate adjusted correlation = 0.85) (Figure 1A,B, Supplementary Tables S1 and S2). FMRP expression levels measured by MSD and PrimeFlow^TM^ negatively correlated with CGG repeat number (*p* = 0.001, covariate adjusted correlation of −0.65, and *p* = 0.02, covariate adjusted correlation of −0.57, respectively) in subjects with fewer than 200 CGG repeats after correcting for percent methylation (Figure 1C,D, Supplementary Tables S3 and S4).

### 3.2. FMRP Expression Levels in PMBCs Correlate with IQ

**Figure 2:**
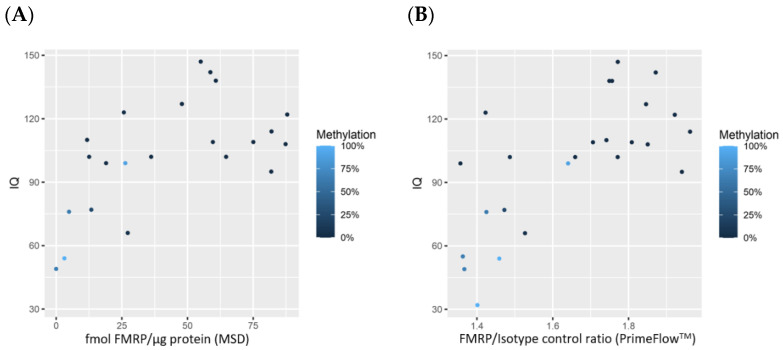
Scatterplot of FMRP expression measured by MSD (*n* = 22) (**A**) and by Flow Cytometry (*n* = 25) (**B**) by IQ. Points are colored by % methylation, with lighter blue corresponding to more methylation and darker blue to less methylation. A small amount of random jitter is applied to the x-axis in order to better display overlapping points.

FMRP expression levels measured by MSD (*p* = 0.004, correlation = 0.59) and by PrimeFlow^TM^ (*p* < 0.001, correlation = 0.69) showed positive correlation with IQ (Figure 2A,B, Supplementary Tables S5 and S6). However, IQ did not significantly correlate with *FMR1* mRNA levels measured by either qRT-PCR or PrimeFlow^TM^ (Supplementary Figure S3A,B, Supplementary Tables S7 and S8).

### 3.3. Correlation of Methods to Measure FMRP and FMR1 mRNA Expression Levels

All assay methods have floors limiting the ability to measure low expression. Non-specific signal and instrument sensitivity are two major components establishing the assay’s floor. The detection floor for *FMR1* mRNA and FMRP in the PrimeFlow^TM^ assay was determined using the dapB probe and an isotype control, respectively. For FMRP detection in the MSD assay, the floor was the lower limit of quantification (LLOQ), 0.65 fmol FMRP per reaction. The floors of these assays contribute to the clustering of data points around the plots’ origins.

**Figure 3:**
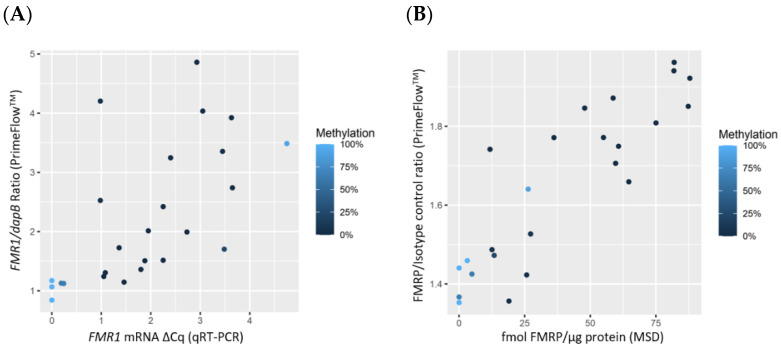
Scatterplots comparing methodologies to measure *FMR1* (*n* = 25) (**A**) and FMRP (*n* = 24) (**B**) expression. Points are colored by % methylation, with lighter blue corresponding to more methylation and darker blue to less methylation. A small amount of random jitter is applied to the x-axis in order to better display overlapping points.

Immunophenotyping was included in the PrimeFlow^TM^ assay to determine if a specific subtype of PBMC expressed FMRP or *FMR1* and if measurement of a PBMC subtype would provide better accuracy. Supplementary Figure S4A–E outlines the analytical approach applied to the flow cytometric data. The results show no subtype of PBMC is a predominant expressor of *FMR1* or FMRP. However, as this study was limited to cross-sectional analysis, FMRP expression may fluctuate in distinct blood cell types over time. The qRT-PCR and PrimeFlow^TM^ techniques to quantify *FMR1* had a moderate correlation of 0.64 (*p* = 0.001) (Figure 3A). In comparison, both approaches used to quantify FMRP expression strongly correlated with one another (correlation of 0.88; *p* < 0.001) (Figure 3B).

### 3.4. Relationship between FMRP and FMR1 Expression Levels

**Figure 4:**
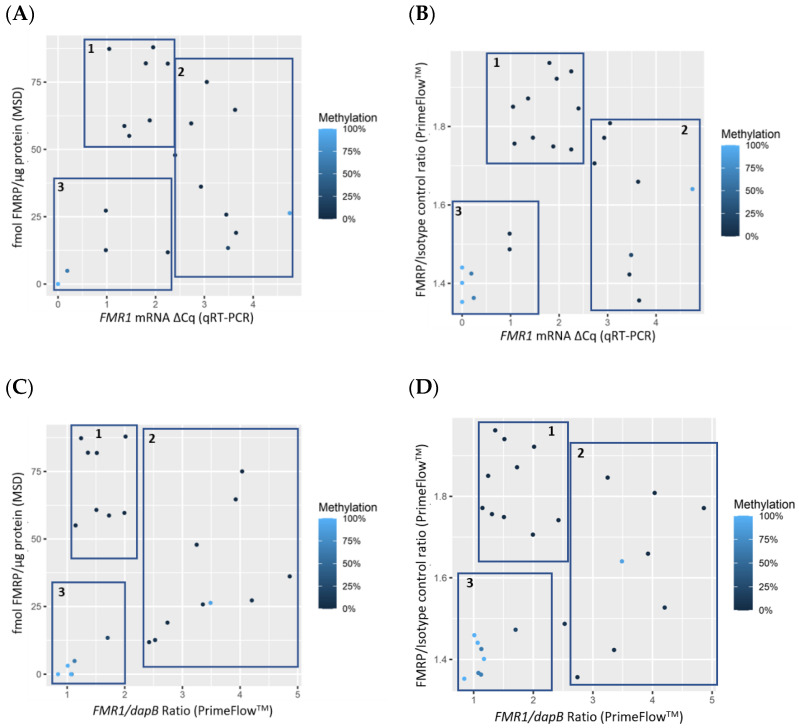
Scatterplots comparing *FMR1* and FMRP expression in PBMCs. (**A**) (*n* = 22) compares *FMR1* mRNA measured by qRT-PCR to FMRP measured by MSD. (**B**) (*n* = 25) is a comparison of *FMR1* mRNA measured by qRT-PCR to FMRP measured by PrimeFlow^TM^. (**C**) (*n* = 24) compares *FMR1* mRNA measured by PrimeFlow^TM^ to FMRP measured by MSD. (**D**) (*n* = 27) is a comparison of *FMR1* mRNA measured by PrimeFlow^TM^ to FMRP measured by PrimeFlow^TM^. Regardless of the methods used to measure *FMR1* mRNA and FMRP, the data graphs into three groups. Group 1: low *FMR1* and high FMRP. Group 2: increasing *FMR1* with decreasing FMRP and decreasing *FMR1* with decreasing FMRP. Group 3: low *FMR1* and low FMRP. Points are colored by % methylation, with lighter blue corresponding to more methylation and darker blue to less methylation. A small amount of random jitter is applied to the x-axis in order to better display overlapping points.

Based on visual examination, FMRP expression levels appeared to be quadratically related to *FMR1* mRNA expression levels. Therefore, a quadratic term for *FMR1* mRNA was included in the model for these data; p-values are reported for a joint test of the linear and quadratic terms. FMRP expression levels measured by both MSD and PrimeFlow^TM^ significantly correlated to *FMR1* mRNA levels measured by qRT-PCR. The quadratic model resulted in *p*-values of 0.010 (MSD) and <0.001(PrimeFlow^TM^) (Figure 4A,B, Supplementary Tables S9 and S10). However, when FMRP was measured by PrimeFlow^TM^, the relationship with *FMR1* mRNA was not significant. The quadratic model resulted in p-values of 0.451(MSD) and 0.420 (PrimeFlow^TM^) (Figure 4C,D, Supplementary Tables S11 and S12).

Three groups of data points were observed. Normal and lower CGG repeat premutation individuals fell into group 1, consisting of normal *FMR1* mRNA and high FMRP expression (*n* = 9). As the number of CGG repeats increased, individuals fell into group 2 (*n* = 10), with increasing *FMR1* mRNA and decreasing FMRP levels. However, lower *FMR1* mRNA expression and FMRP were observed for those who had some cells carrying methylated alleles and therefore were transcriptionally silent. Individuals with a hypermethylated full mutation, except for 1 methylation mosaic, fell into group 3 with low *FMR1* mRNA and low FMRP (*n* = 8) (Figure 4A–D). At the time of analysis, blinding to the CGG repeat length and methylation status was maintained. Table 1 indicates which group each participant was assigned.

## 4. Discussion

FMRP is an RNA binding protein required for normal synaptic maturation and dendritic pruning, as its absence leads to altered dendritic spine density, size, and shape. Consequently, immature synapses, which have been reported in both mice and humans [35], result in neuronal dysfunction and ultimately in the cognitive and behavioral deficits observed in FXS. A relationship between FMRP expression and the size and volume of brain regions has been reported in FXS, supporting the hypothesis that lack of FMRP leads to an abnormal neuronal organization within brain regions, particularly of those involved in response inhibition [36]. A recent report by Schneider et al. [37] also found a correlation between lower FMRP expression and psychotic features in FXS; and interestingly, between FMRP deficits and lower IQ in individuals without the *FMR1* mutation who present with schizophrenia [38] or other neuropsychiatric disorders, including autism. These findings indicated the wider importance of targeting FMRP-deficiency-based mechanisms [39]. Further, an overwhelming preponderance of evidence gathered in the past ten to fifteen years has demonstrated a strong relationship between the lack of FMRP with both general intellectual impairment and specific cognitive functioning deficits observed in both males and females with the fragile X full mutation [22,23,40,41,42,43,44,45,46]. Although it was reported that partial restoration of FMRP, greater than 20%, was sufficient to normalize neuronal activity in FXS iPSC-derived neurons5, the association between the range of FMRP expression and the severity of developmental and cognitive disabilities is still not well understood.

In a recent paper, Kim and colleagues [23] reported on the association between FMRP levels, measured in fibroblast cell lines, and IQ in 184 individuals with the *FMR1* mutation spanning from normal to full mutation. They found that within the normal CGG repeat range, IQ is not affected by FMRP levels above the threshold of ~70% of the mean. Interestingly they also observed that ~35% of the mean FMRP level measured in the normal CGG repeat range was of a sufficient magnitude to result in a mean IQ of 85. However, their cohort included 20 participants with a full mutation (mosaics and non-mosaics) but no data on the degree of methylation (percent of cells carrying unmethylated alleles or activation ratio in females) or CGG repeat number, so it was not clear if the data were corrected to take these variables into account. 

In another study [47], FMRP levels were measured in a reference set of cell lines, blood, and buccal specimens with a range of *FMR1* CGG repeats expansions. Although mostly focusing on those with FXS, their data showed an inverse relationship between overall severity of the FXS phenotype and reduced FMRP levels. However, substantial FMRP expression was observed in a hypermethylated full mutation male derived sample which the authors attributed to “undetected” epigenetic mosaicism. Mosaicism is quite common in FXS [19], and it results in differential transcriptional and translational expression of the *FMR1* gene in neurons and other cell types; however, the degree of gene expression depends on the CGG repeat number and on the percent of cells carrying unmethylated alleles. Although *FMR1* unmethylated alleles, even in the full mutation range, [16] are transcribed, they are not efficiently translated into FMRP starting from the upper premutation into the full mutation range [18,48]. Thus, the lower FMRP expression detected in individuals with a full mutation and mosaicism [20,21,41,49] and in individuals carrying a premutation allele [20,21,50] could be responsible for the clinical, cognitive, and behavioral impairment seen in fragile X syndrome and *FMR1* associated disorders [14,26]. 

A recent survey involving family members and caregivers of FXS affected individuals revealed the three main areas of concern to be anxiety, behavioral problems, and learning difficulties [51]. Therefore, since cognitive ability positively correlates to expression of FMRP in PBMCs (which are the most easily accessible human tissue for repeat collection), it is important to correctly measure FMRP expression in PBMCs. To this regard, robust and reliable FMRP assays that can be applied directly to PBMCs for future trials of therapeutic reactivation of FMRP are still clearly needed.

Among the different methodologies reported to detect and quantify FMRP levels are Western blot, immunohistochemistry, ELISA, and most recently FRET. However, none of these formats have been proven, so far, to work efficiently, reliably, and specifically in PBMCs. Thus, the need for a quantitative, high throughput assay with a low false discovery rate and lower limit of detection below reported assays that can be easily transferred to other laboratories remains. Immunohistochemistry is mostly qualitative and requires a highly skilled individual to execute the assay and discern positive from negative samples without bias. Immunohistochemical approaches have high false positive and negative rates and are not easily transferred between labs. Although Western blots are mostly semi-quantitative, Lessard et al [52] reported on a quantitative Western blot employing the Odyssey (LI-COR) fluorescence Western blot system using lysates from isolated platelets. Lessard reported the FMRP mean for non-affected controls was 29.6 ± 7.5 pg/106 platelets. The lowest detectable level of FMRP in an affected male was 8.6 pg/106 platelets. Although the study quantified FMRP from Western blot bands on the Odyssey, use of a single anti-FMRP antibody, variations in transfer efficiency and variability in blot exposure time can lead to low sensitivity and potentially high false positive and negative rates.

Assays such as ELISAs, Luminex, and FRET are more quantitative, have lower error, and are more easily transferred between labs compared to the previously mentioned assays. The use of two anti-FMRP antibodies in these assays improves selectivity and reduces the rate of false positives. In 2009, Iwahashi et al [53] developed a quantitative ELISA to detect FMRP from isolated lymphocyte lysates. The sensitivity of the ELISA was 5pM per reaction. Recently, FMRP levels in isolated, cultured fibroblasts were reported using a high throughput time resolved FRET (HTRF) assay (Cisbio, Bedford, MA, USA). However, the authors reported only relative quantification values of FMRP as standard deviations from the mean of fibroblasts isolated from individuals with a normal CGG repeat number. Our lab tested the Cisbio HTRF assay to determine the LLOD by utilizing a full length recombinant FMRP (rFMRP). The LLOD of the HTRF assay was 7 fmol FMRP per reaction. The HTRF assay was further investigated by running cellular lysates. The lowest amount of FMRP detected from the cellular lysates was 85 fmol FMRP/μg of total protein. Given the numerous types of FMRP detection assays and the varied data reporting strategies, it is difficult to compare sensitivity and reproducibility across the reported methods.

In the presented study, two novel immunoassays to measure *FMR1* mRNA and FMRP expression directly in PBMCs were reported. One is an FMRP specific, electrochemiluminescent MSD assay, which exhibited an LLOD of 0.07 fmol and an LLOQ of 0.65 fmol FMRP per reaction. The other is a multiparameter, flow cytometric assay with the ability to simultaneously determine the relative expression of *FMR1* mRNA and FMRP on a cell-by-cell basis. These assays were used to determine *FMR1* mRNA and FMRP expression levels in PBMCs derived from participants with different *FMR1* CGG allele sizes and methylation status. Significant correlations were observed between the different methods to measure FMRP and *FMR1* mRNA expression levels which confirmed our and other previous studies. Importantly, FMRP expression levels, as detected by either MSD or PrimeFlow^TM^ in PBMCs, had a robust positive correlation with IQ.

The observed correlation between the two FMRP methods was stronger than the relationship between the *FMR1* mRNA methods for several potential reasons: (1) qRT-PCR requires a reverse transcription step to convert mRNA to cDNA whereas PrimeFlow^TM^ is a direct measurement of mRNA, (2) slight differences in the probes used by each method to detect *FMR1* mRNA expression, and (3) the qRT-PCR results are reported as relative to a housekeeping gene vs. the PrimeFlow^TM^ in which results are a ratio to background (dapB). In contrast, the detection of FMRP by MSD and PrimeFlow^TM^ use similar anti-FMRP antibodies to directly detect FMRP, which may explain the stronger correlation.

In conclusion, this study is of importance because the methods developed are sensitive and accurate to quantify FMRP, do not require many cells, are rapid and cost-effective, and can be easily implemented in most laboratories. Of extreme relevance is that this method works in blood (PBMCs); sampling the blood is minimally invasive and avoids the complications of biopsies or of other collection approaches. These accurate FMRP measurements will be helpful in assessing the degree of FMRP deficiency in individuals with an expansion throughout the CGG repeat range (normal to full mutation) using readily obtainable clinical samples, in different tissues and developmental stages. 

Finally, both the MSD and PrimeFlow^TM^ methods could be used in prospective clinical trials to assess the role of FMRP as a biomarker of treatment efficacy.

## Figures and Tables

**Table 1:** Study Participants and Measurements Acquired.

Case Number	Age (yrs)	Category	CGG Repeat	% Methylation *	IQ	IQ Test	*FMR1* (qRT-PCR)	MSD FMRP (fmol/μg Protein)	PrimeFlow^TM^ Relative FMRP	PrimeFlow^TM^- Relative *FMR1*	*FMR1*/FMRP Group **
1	72.5	Normal	30		147	WAIS-3	1.88	55.03	1.77	1.15	1
2	51.9	Normal	30		138	WAIS-3	1.08	60.78	1.75	1.51	1
3	68.9	Normal	20		138	WAIS-3	1.05	Not Tested	1.76	1.31	1
4	65.6	Normal	26		108	WASI II	2.73	87.32	1.85	1.24	1
5	55.3	Premutation	89		109	WAIS-4	2.25	59.66	1.71	1.99	1
6	59.8	Premutation	30–200		110	WAIS-4	2.25	11.78	1.74	2.42	2
7	47.7	Premutation	90		95	WASI II	1.95	81.86	1.94	1.52	1
8	74.5	Premutation	74		122	WASI II	3.05	87.94	1.92	2.01	1
9	49.9	Premutation	123		109	WAIS III	3.63	75.04	1.81	4.04	2
10	14.4	Premutation	133		102	Standard Binet	1.8	64.69	1.66	3.92	2
11	71.8	Premutation	63		114	WAIS-4	1.36	81.96	1.96	1.36	1
12	50	Premutation	57		142	WAIS-3	2.93	58.70	1.87	1.73	1
13	55.4	Premutation	141		102	WAIS-3	2.4	36.16	1.77	4.86	2
14	66.9	Premutation	107		127	WAIS-4	3.65	47.87	1.85	3.25	2
15	7.9	Premutation/meth	150, 180	2	99	WISC-IV	3.45	19.01	1.36	2.74	2
16	7.9	Premutation/meth	157, 180	3	123	WISC-IV	4.75	25.77	1.42	3.36	2
17	19.7	Premutation/meth	177	90	99	WAIS-4	0.98	26.32	1.64	3.49	2
18	18.6	Premutation/meth	156	5	66	WAIS-4	0	27.26	1.53	4.20	2
19	19	Full mutation	>200	100	32	Leiter	0	Not Tested	1.40	1.17	3
20	15.2	Full mutation	>200	100	NA	NA	0	Below LLOQ	1.35	0.84	3
21	21.4	Full mutation	>200	100	NA	NA	NA	Below LLOQ	1.44	1.07	3
22	10.4	Full mutation	>200	100	54	Leiter	0.24	3.14	1.46	1.01	3
23	13.5	Meth Mosaic	>200 (~215)	65	55	Standard Binet	NA	Not Tested	1.36	1.12	3
24	3.4	Meth mosaic	>200 (360–530)	65	49	MSEL	3.48	Below LLOQ	1.37	1.08	3
25	36.3	Meth mosaic	>200 (unmethylated smear)	23	77	Standard Binet	0.19	13.38	1.47	1.70	3
26	3.2	Meth Mosaic	>200 (330–470)	62	76	Leiter	0.98	4.90	1.43	1.13	3
27	36.9	Meth Mosaic	~200 (normal-670)	5	102	WAIS-4	NA	12.57	1.49	2.53	2

* All samples tested for methylation. Cells are blank if no methylation was detected; ** Groups are as follow: group 1: low *FMR1* and high FMRP; group 2: increasing *FMR1* with decreasing FMRP and decreasing *FMR1* with decreasing FMRP; group 3: low *FMR1* and low FMRP. Methylation is abbreviated as meth; lower limit of quantification is abbreviated as LLOQ.

## Data Availability

Data used for statistical analysis and to generate Figure 1, Figure 2, Figure 3 and Figure 4 can be found in Table 1. Additional data are available upon request.

## Supplementary Materials

The following are available online at https://www.mdpi.com/article/10.3390/diagnostics11101780/s1, Figure S1: Typical MSD ELISA rFMRP standard curve, Figure S2: Typical workflow for running the PrimeFlow^TM^ and MSD assays on isolated, frozen PBMCs, Figure S3: Scatterplot of *FMR1* expression measured by qRT-PCR (A) or PrimeFlow^TM^ (B) by IQ, Figure S4: PrimeFlow^TM^ quantification of *FMR1* and FMRP with simultaneous immunophenotyping, Table S1: Linear Model of *FMR1* (qRT-PCR) by CGG, Table S2: Linear Model of *FMR1* (PrimeFlow^TM^) by CGG, Table S3: Linear Model of FMRP (MSD) by CGG, Table S4: Linear Model of FMRP (PrimeFlow^TM^) by CGG, Table S5: Linear Model of IQ by FMRP (MSD), Table S6: Linear Model of IQ by FMRP (PrimeFlow^TM^), Table S7: Linear Model of IQ by *FMR1* (qRT-PCR), Table S8: Linear Model of IQ by *FMR1* (PrimeFlow^TM^), Table S9: Quadratic Model of FMRP (MSD) by *FMR1* (qRT-PCR), Table S10: Quadratic Model of FMRP (PrimeFlow^TM^) by *FMR1* (qRT-PCR), Table S11: Quadratic Model of FMRP (MSD) by *FMR1* (PrimeFlow^TM^), Table S12: Quadratic Model of FMRP (Flow) by *FMR1* (PrimeFlow^TM^).

## Abbreviations

ADHD	Attention-deficit/Hyperactivity Disorder
ASD	Autism Spectrum Disorders
AUC	Area Under the Curve
BCA	Bicinchoninic Acid
CPT	Cell Preparation Tube
dapB	4-hydroxy-tetrahydrodipicolinate reductase gene
*FMR1*	Fragile X Mental Retardation Gene
FMRP	Fragile X Mental Retardation Protein
FRET	Fluorescence Resonance Energy Transfer
FXAND	Fragile X-associated Neuropsychiatric Disorders
FXPOI	Fragile X-associated primary ovarian insufficiency
FXS	Fragile X Syndrome
FXTAS	Fragile X-associated tremor/ataxia syndrome
HTRF	High Throughput Time Resolved FRET
iPSC	Induced Pluripotent Stem Cells
IQ	Intelligence Quotient
LLOD	Lower Limit of Detection
LLOQ	Lower Limit of Quantification
MSD	MesoScale Discovery
MSEL	Mullen Scales of Early Learning
PBMCs	Peripheral Blood Mononuclear Cells
PBS	Phosphate Buffered Saline
PCR	Polymerase Chain Reaction
UTR	Untranslated region
WASI	Weschler abbreviated Scale of Intelligence
WAS-III	Wechsler Intelligence Scale for Children
