# Supplementary material for: FMRP Levels in Human Peripheral Blood Leukocytes Correlates with Intellectual Disability
Source: Diagnostics (Basel). 2021 Sep 28;11(10):1780. doi: 10.3390/diagnostics11101780 (PMC8534530; doi:10.3390/diagnostics11101780)
Supplement: Supplementary file 1 [file diagnostics-11-01780-s001.zip › diagnostics-1324800-supplementary.pdf]

Supplementary figures and tables: FMRP levels in human peripheral blood leukocytes correlates with intellectual disability

Supplementary figure S1: Typical MSD ELISA rFMRP standard curve. The LLOD and LLOQ are indicated. Data from a full mutation, a premutation, and a normal control with equivalent lysate protein concentrations are included for illustration.

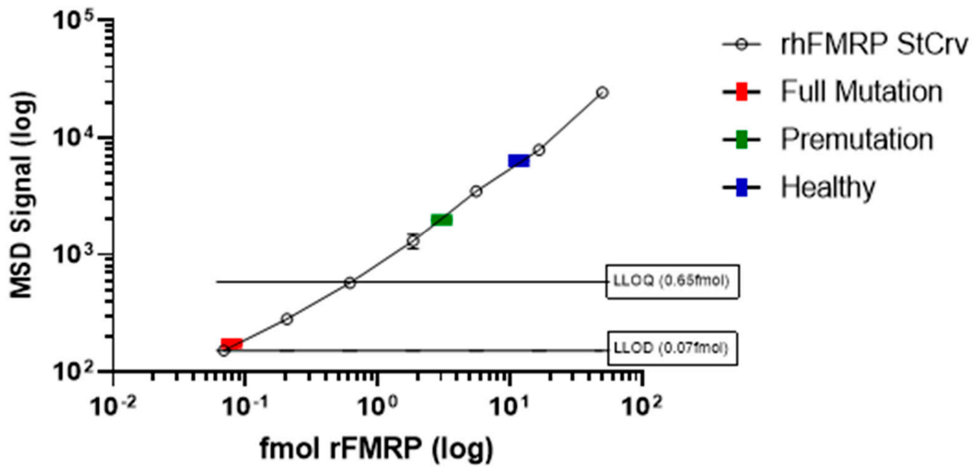

Supplementary figure S2: Typical workflow for running the PrimeFlow™ and MSD assays on isolated, frozen PBMCs. The PrimeFlow™ and MSD are 2-day assays

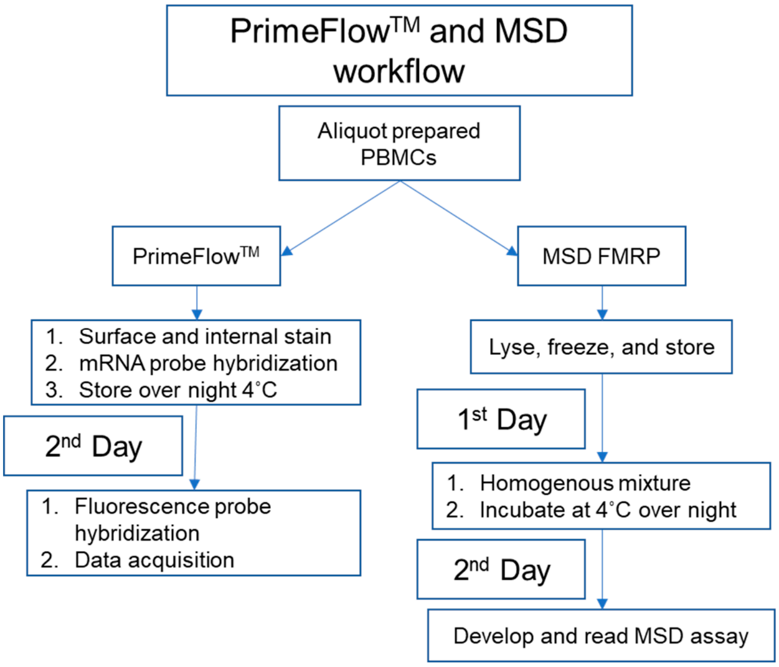

Supplementary table S1: Linear Model of *FMR1* (qRT-PCR) by CGG

	**Estimate (95% CI)**	**P-Value**
Slope (CGG < 200)	0.017 (0.007, 0.026)	0.00142
Mean (CGG >= 200)	0.979 (0.052, 1.907)	0.03952

Adjusted R-Squared = 0.533

[a] % methylation was included as a covariate.

[b] Slope = change in *FMR1* expression for a one-repeat increase in CGG repeat length, for subjects with fewer than 200 CGG repeats

[c] Mean = Mean *FMR1* expression for subjects for 200 or more CGG repeats, assuming methylation equal to the study mean

Supplementary table S2: Linear Model of *FMR1* (PrimeFlow™) by CGG

	Estimate (95% CI)	P-Value
Slope (CGG < 200)	0.022 (0.016, 0.027)	<0.001
Mean (CGG >= 200)	1.778 (1.238, 2.317)	<0.001

Adjusted R-Squared = 0.777

[a] % methylation was included as a covariate.

[b] Slope = change in *FMR1* expression for a one-repeat increase in CGG repeat length, for subjects with fewer than 200 CGG repeats

[c] Mean = Mean *FMR1* expression for subjects for 200 or more CGG repeats, assuming methylation equal to the study mean

Supplementary table S3: Linear Model of FMRP (MSD) by CGG

	Estimate (95% CI)	P-Value
Slope (CGG < 200)	-0.335 (-0.52, -0.151)	0.00115
Mean (CGG >= 200)	8.284 (-8.591, 25.159)	0.31808

Adjusted R-Squared = 0.714

[a] % methylation was included as a covariate.

[b] Slope = change in FMRP expression for a one-repeat increase in CGG repeat length, for subjects with fewer than 200 CGG repeats

[c] Mean = Mean FMRP expression for subjects for 200 or more CGG repeats, assuming methylation equal to the study mean

Supplementary table S4: Linear Model of FMRP (PrimeFlow™) by CGG

	Estimate (95% CI)	P-Value
Slope (CGG < 200)	-0.002 (-0.003, -0.001)	0.00286
Mean (CGG >= 200)	1.427 (1.312, 1.542)	< 0.001

Adjusted R-Squared = 0.672

[a] % methylation was included as a covariate.

[b] Slope = change in FMRP expression for a one-repeat increase in CGG repeat length, for subjects with fewer than 200 CGG repeats

[c] Mean = Mean FMRP expression for subjects for 200 or more CGG repeats, assuming methylation equal to the study mean

Supplementary table S5: Linear Model of IQ by FMRP (MSD)

	Estimate (95% CI)	P-Value
Slope	0.517 (0.184, 0.85)	0.00408
	Adjusted R-Squared = 0.312	

[a] Slope = change in IQ for a unit increase in FMRP

Supplementary table S6: Linear Model of IQ by FMRP (PrimeFlow™)

	Estimate (95% CI)	P-Value
Slope	103.875 (56.342, 151.409)	<0.001
	Adjusted R-Squared = 0.447	

[a] Slope = change in IQ for a unit increase in FMRP

Supplementary figure S3: Scatterplot of *FMR1* expression measured by qRT-PCR (A) or PrimeFlow™ (B) by IQ. Points are colored by % methylation, with lighter blue corresponding to more methylation and darker blue to less methylation. A small amount of random jitter has been applied to the x-axis in order to better display overlapping points.

A B

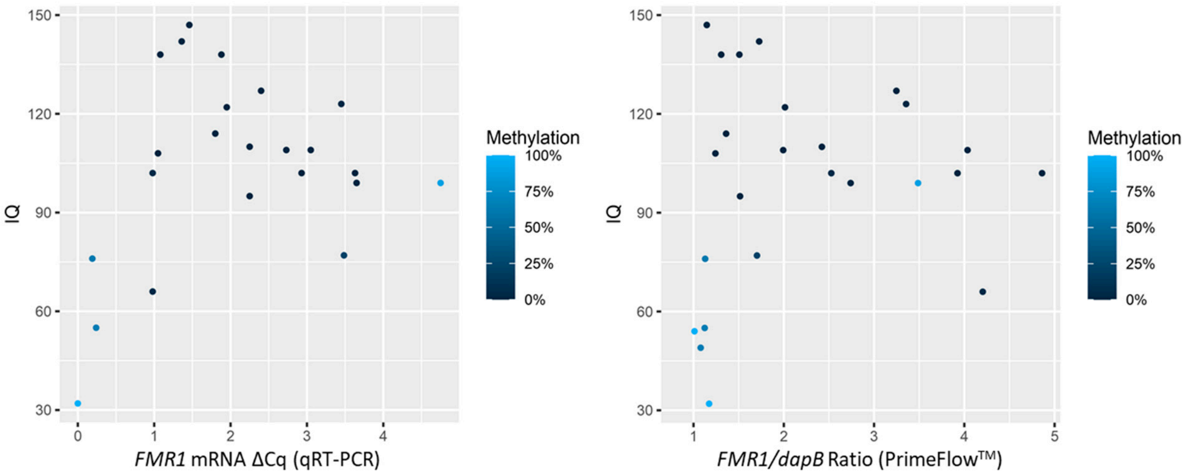

Supplementary Table S7: Linear Model of IQ by *FMR1* (qRT-PCR)

	Estimate (95% CI)	P-Value
Slope	3.054 (-4.6, 10.709)	0.415

Adjusted R-Squared = 0.439

[a] Slope = change in IQ for a unit increase in *FMR1* expression

[b] Percent methylation was included as a covariate.

Supplementary Table S8: Linear Model of IQ by *FMR1* (PrimeFlow™)

	Estimate (95% CI)	P-Value
Slope	-2.899 (-10.895, 5.097)	0.46

Adjusted R-Squared = 0.539

[a] Slope = change in IQ for a unit increase in *FMR1* expression

[b] Percent methylation was included as a covariate.

Supplementary figure S4: PrimeFlow™ quantification of *FMR1* and FMRP with simultaneous immunophenotyping. (A) Discrimination of CD19^{+} B-cells and CD3^{+} T-cells. (B) The CD3^{+} T-cells are further subdivided into CD3^{+}CD4^{+} T-helper and CD3^{+}CD8^{+} T-cytotoxic cells. (C) Determined CD14^{+} monocyte subpopulation excluding CD3+ cells. (D) Example of *FMR1* identification in monocytic population. (E) Example of FMRP identification in T-helper population. The average *FMR1* MFI / *dapB* MFI ratio of the 4 cellular populations are reported as the *FMR1* relative quantification. The average FMRP MFI / IC control MFI ratio of the 4 cellular populations are reported as the FMRP relative quantification.

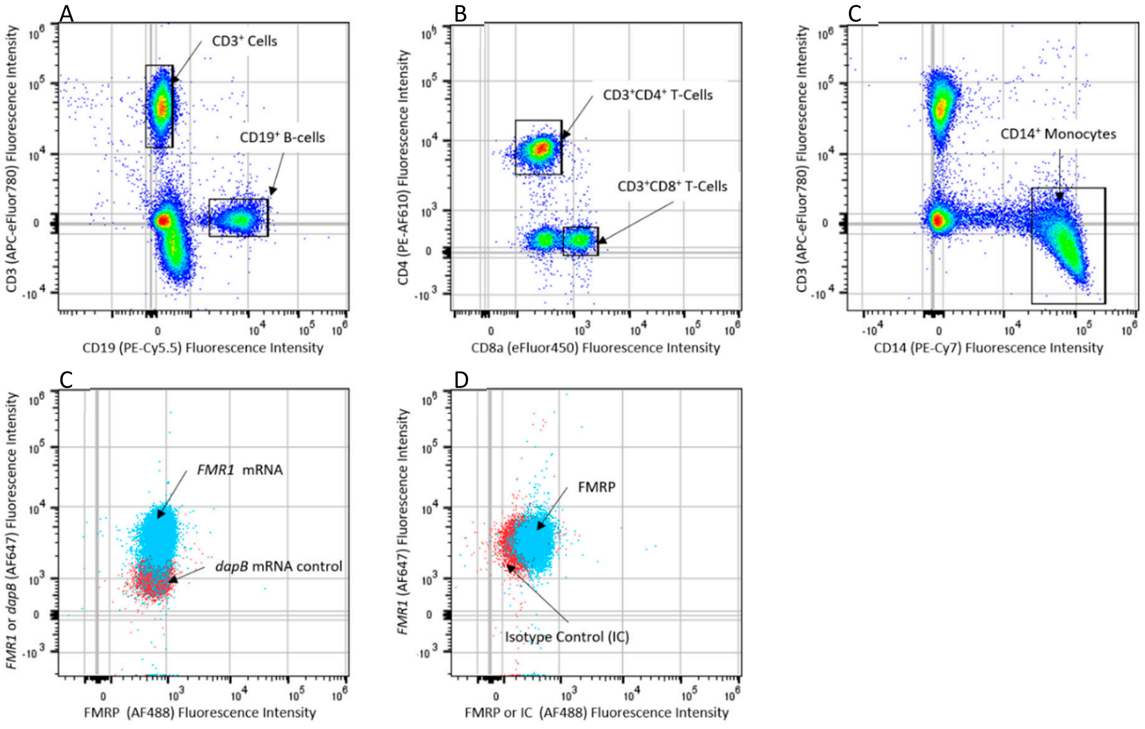

Supplementary table S9: Quadratic Model of FMRP (MSD) by *FMR1* (qRT-PCR)

	**Estimate (95% CI)**	**P-Value**
Linear Term	45.57 (17.83, 73.31)	0.00275
Quadratic Term	-9.709 (-15.867, -3.55)	0.00377
	Adjusted R-Squared = 0.319	

Supplementary Table S10: Quadratic Model of FMRP (PrimeFlow™) by *FMR1* (qRT-PCR)

	**Estimate (95% CI)**	**P-Value**
Linear Term	0.336 (0.185, 0.487)	<0.001
Quadratic Term	-0.074 (-0.109, -0.038)	<0.001
	Adjusted R-Squared = 0.445	

Supplementary table S11: Quadratic Model of FMRP (MSD) by *FMR1* (PrimeFlow™)

	Estimate (95% CI)	P-Value
Linear Term	25.324 (-36.361, 87.009)	0.403
Quadratic Term	-4.225 (-15.673, 7.223)	0.451
	Adjusted R-Squared = -0.051	

Supplementary table S12: Quadratic Model of FMRP (Flow) by *FMR1* (PrimeFlow™)

	Estimate (95% CI)	P-Value
Linear Term	0.184 (-0.212, 0.58)	0.346
Quadratic Term	-0.03 (-0.104, 0.045)	0.420
	Adjusted R-Squared = -0.024	
